# Relation of sedentary behaviour to physical function in phase I cardiac rehabilitation

**DOI:** 10.1038/s41598-023-36593-4

**Published:** 2023-06-09

**Authors:** Kazuhiro P. Izawa, Yuji Kanejima, Masahiro Kitamura, Kodai Ishihara, Asami Ogura, Ikko Kubo, Koichiro Oka, Hitomi Nagashima, Hideto Tawa, Daisuke Matsumoto, Ikki Shimizu

**Affiliations:** 1 Department of Public Health, Graduate School of Health Sciences, Kobe University, 10-2 Tomogaoka 7-Chome, Suma-Ku, Kobe, 654-0142 Japan; 2 Cardiovascular Stroke Renal Project (CRP), Kobe, Japan; 3 Department of Rehabilitation, Kobe City Medical Center General Hospital, Kobe, Japan; 4 School of Physical Therapy, Faculty of Rehabilitation, Reiwa Health Sciences University, Fukuoka, Japan; 5 Department of Physical Therapy, Faculty of Nursing and Rehabilitation, Konan Women’s University, Kobe, Japan; 6 Department of Rehabilitation, Sanda City Hospital, Sanda, Japan; 7 Department of Rehabilitation, Yodogawa Christian Hospital, Osaka, Japan; 8 Faculty of Sport Sciences, Waseda University, Saitama, Japan; 9 Department of Rehabilitation, Shinyukuhashi Hospital, Yukuhashi, Japan; 10 Department of Cardiology, Sanda City Hospital, Sanda, Japan; 11 Department of Cardiovascular Medicine, Yodogawa Christian Hospital, Osaka, Japan; 12 Department of Diabetes, Sakakibara Heart Institute of Okayama, Okayama, Japan

**Keywords:** Environmental sciences, Cardiology, Health care, Health occupations

## Abstract

Increased sedentary behaviour (SB) is reportedly associated with mortality and morbidity in cardiovascular disease. However, its relation with physical function is not well understood in phase I cardiac rehabilitation (CR). This study aimed to investigate the rate of SB and the relation between SB and physical function among patients participating in phase I CR. This prospective multicentre cohort study enrolled patients participating in CR from October 2020 to July 2022. Patients with probable dementia and difficulty walking alone were excluded. We used sitting SB time as the index of SB and the Short Performance Physical Battery (SPPB) as the index of physical function at discharge. Patients were divided into the low SB group (< 480 min/day) or high SB group (≥ 480 min/day). We analysed and compared the two groups. The final analysis included 353 patients (mean age: 69.6 years, male: 75.6%), of whom 47.6% (168 of 353) were high SB patients. Total sitting SB time was higher in the high SB group versus the low SB group (733.6 ± 155.3 vs 246.4 ± 127.4 min/day, p < 0.001), and mean SPPB score was lower in the high SB group versus the low SB group (10.5 ± 2.4 vs 11.2 ± 1.6 points, p = 0.001). Multiple regression analysis identified SB as an explanatory variable for total SPPB score (p = 0.017). Patients with high SB had significantly lower SPPB scores than those with low SB. These findings underscore the importance of considering SB when improving physical function. Effective strategies to improve physical function can be developed that consider SB in phase I CR.

## Introduction

Patients with cardiovascular disease (CVD) such as heart failure (HF), ischaemic heart disease, and valvular disease typically report low levels of physical activity, which affect health-related quality of life (HRQOL) and increase the risk of disease progression and mortality^1–3^. The promotion of physical activity (exercise, sports, and increased activities of daily living) and exercise behaviour are effective in preventing or improving various diseases such as diabetes, obesity, some cancers, and high blood pressure^4–7^. Despite the regular promotion of physical activity, recent research in the USA and Europe has found increased health risks for death, obesity, diabetes, and CVD in people with sedentary behaviour (SB; i.e., any waking behaviour characterized by an energy expenditure of ≤ 1.5 metabolic equivalents while sitting, reclining, or lying down), and SB could be a potential target for improving cardiovascular health^4,8^. Moreover, it has been known for a long time that sitting SB time per day in the Japanese adult population is higher than that in foreign countries^9^. Thus, it is important not only to promote physical activity in the Japanese lifestyle but also to decrease sitting SB time to improve health.

Several previous reports have shown that in hospitalized patients, physical activity such as standing and/or walking decreased to 36% or less per day during their hospitalization, that they spent most of their time in bed or sitting, and that increased comorbidities were associated with a lower daily number of steps and lower upright time^10–13^. Patients with CVD were also reported to have greater amounts of objectively measured sedentary time compared to healthy controls^14^, and sedentarism was associated with personal and lifestyle characteristics and comorbidities^14,15^. Another report also suggested high levels of SB (9.7 ± 2.0 h/day) among patients with CVD at 28 days after discharge^16^.

The Short Physical Performance Battery (SPPB) is a series of tests used together to measure the functional performance of the lower extremities^17–19^. The test consists of three major components, each of which are scored independently: a set of 3 static balance tests, measurement of gait speed, and the five time sit-to-stand test^17–19^. The SPPB is now frequently being used as a screening tool for sarcopenia in elderly patients with CVD^17–19^. As the SPPB does not require special procedures or skills in evaluation, physical function and sarcopenia can be easily evaluated even in elderly people with complications^18,19^. For these reasons, the SPPB was used to assess physical function and perform SB screening in the present study.

Previous studies have also shown that 63.8% of elderly patients with HF had low physical function as assessed by the SPPB at discharge^18^ and that the motor Functional Independence Measure score of activities of daily living in these patients was an independent predictor of re-hospitalization within 90 days^20^. Another recent report found that the prevalence of hospital-associated disability was 7.43% among 238,160 patients with acute HF eligible for cardiac rehabilitation (CR), and only 44% patients received phase I CR (pre discharge) in the total cohort^21^. Comprehensive CR is composed of exercise and education programs that improve exercise capacity, HRQOL, and prognoses related to rates of mortality and readmission^1–3^, and such programs that included patient education reduced mortality by 73% compared to exercise-only programs^1^. In addition, SB is a potentially important target and may predict gains in cardiac knowledge received from a patient education program in phase II CR (post discharge)^14,16^. Thus, assessment of SB may also be necessary for phase II CR, especially for patients with high SB so that they can better understand and consider the contents of the education they receive during phase II CR.

However, there are few reports on the rate of SB and the relation between SB and the SPPB in patients with CVD participating in phase I CR (pre discharge). We hypothesized that patients with high SB would have poorer physical function as indicated by the SPPB score than patients with low SB. The present study aimed to investigate the rate of SB and the relation between SB and SPPB as an indicator of physical function and to consider further practical interventions for phase I CR.

## Methods

### Study design

This prospective multicentre cohort study included patients from four affiliated small-to-medium-scale hospitals with 200–580 beds, all of which conduct CR. Inclusion criteria comprised patients who were admitted to the affiliated hospitals from October 2020 to July 2022, participated in phase I CR, were hospitalized for more than five days, and had not been hospitalized for a medical procedure such as coronary angiography and percutaneous coronary intervention without CR. Excluded were patients with probable dementia (based on diagnosis or Mini-Mental State Examination score < 24), difficulty walking alone, disagreement with informed consent, hospital death, and data deficits.

### Phase I CR

Comprehensive CR was based on the Japanese Circulation Society guidelines for rehabilitation in patients with CVD^1^. Phase I CR started within three days after admission and cardiac surgery^1,22,23^. The patients in phase I CR performed exercise based on frequency, intensity, type, and time as follows: *frequency*: exercise frequency was encouraged for 5 to 7 days during each week of CR; *intensity*: exercise intensity was encouraged within a Borg scale range of 11 to 13 or just below the patient’s anaerobic metabolism threshold; *type*: exercise types included stretching, aerobic exercise, and resistance training; and *time*: stretching exercises for the upper and lower body were performed for 10 to 20 min, and during aerobic exercise, patients performed warm-up, aerobics, and cool-down exercises for < 25 min, followed by 10 to 20 min of resistance training each day^23^.

Patients were lectured on diseases, lifestyle choices, nutrition, medicines they were taking, and exercise as offered by a doctor, nurse, registered dietitian, pharmacist, and physical or occupational therapists, respectively. Training on these exercises was adjusted according to each patient’s condition.

### Patient characteristics

Patient characteristics such as age, sex, body mass index (BMI), employment, living together, smoking, marriage, main diagnosis, left ventricular ejection fraction, comorbidities, Charlson Comorbidity Index^24^, levels of serum haemoglobin and creatinine, and medications at the time of admission were collected from the medical records.

### Sitting SB time

The Workforce Sitting Questionnaire^25^, whose reliability and validity are already confirmed in Japan^26^, was used as the basis for the questionnaire survey performed in this study to assess sitting SB time. This questionnaire contains six items related to sitting SB time over 1 week that reflect various life scenarios of driving, transportation, work, television viewing, personal computer/smartphone use, and other leisure time activity. Because the patients are hospitalized, driving and transportation times equal zero minutes. Patients were asked to answer each item for workdays and non-workdays over a 7-day (1-week) period. After a researcher gathered up all of the questionnaires, total sitting SB time as it related to the six life scenarios over the 7-day period were calculated. Following that, total sitting SB time in minutes/day was calculated by the researcher as total sitting SB time in minutes/7 days^27,28^. Thus, we considered sitting SB time for the entire day to indicate SB. Then, on the basis of a previous study, we divided the patients into two groups according to a cutoff value for SB of 480 min/day (low SB group: < 480 min/day, high SB group: ≥ 480 min/day)^29^.

### SPPB for physical function

We used the SPPB as an index of physical function to evaluate standing balance, 4-m walking time, and standing/sitting times × 5. The SPPB is scored from 0 points (unable to complete a task) to 4 points (highest level of performance). The sum of the three test scores, which range from 0 to 12 points, was determined according to previously reported methods^18,30^. Patient characteristics were collected by one of the researchers from the medical record on the patient’s admission, and each patient’s SB and SPPB were assessed at discharge by one or two CR staff members.

### Statistical analysis

All analyses were conducted on patients with no missing data for any of the variables. We first calculated the proportion of patients in the high SB group to all patients. We then compared the low SB and high SB groups using an unpaired *t*-test for continuous variables and Pearson’s Chi-squared test for categorical variables. A multiple linear regression analysis was conducted for the total SPPB score as the dependent variable. According to previous studies, we selected independent variables related to SB or SPPB such as age, sex, BMI, employment, smoking, marriage, main diagnosis, comorbidities, and variables that were statistically significant between the two groups^31,32^. Univariable and multivariable models were constructed. R studio (R version 4.0.2) was the analysis software^33^ used, and significance was regard as a p level < 0.05.

### Ethical approval

This study was approved by the institutional review board for ethics at the Graduate School of Health Sciences, Kobe University (Approval no. 951-1), and each affiliated hospital received approval from its local ethics committee.

### Informed consent

Informed consent was obtained from each patient.

## Results

### Patient selection

**Figure 1:**
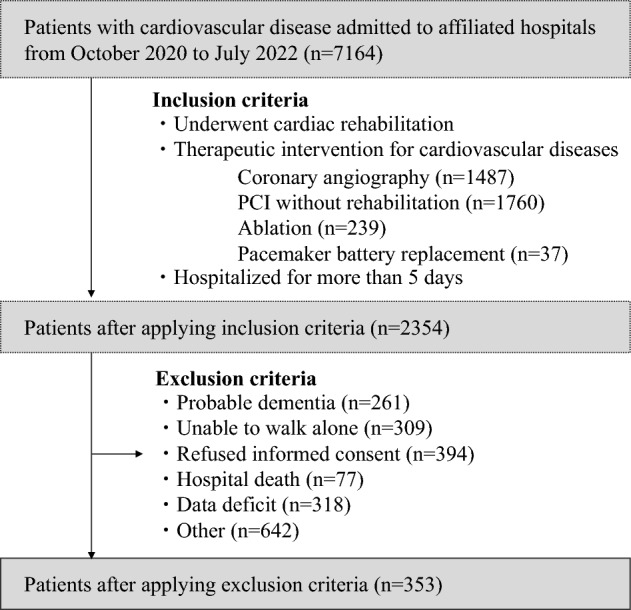
Flow chart of the study participants. *PCI* percutaneous coronary intervention.

Figure 1 shows the flow chart of patients in the study. Among 7164 patients with cardiac disease admitted to the affiliated hospitals during the study period, 2354 patients met the inclusion criteria, including being hospitalized for more than five days and undergoing CR. After further excluding patients, 353 patients (mean age: 69.6, male; 75.6%) were finally included in the present study.

### Rate of SB and comparison between the high and low SB groups

**Figure 2:**
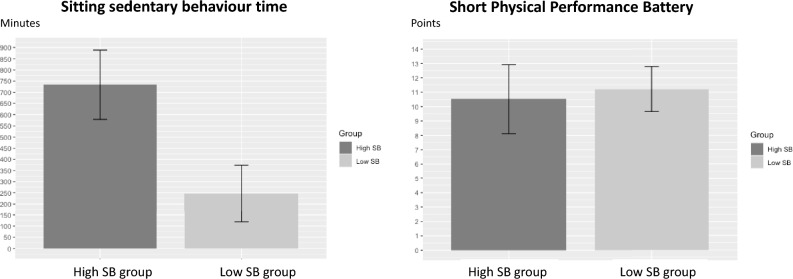
Comparison between the high sedentary behaviour (SB) group and low SB group for sitting SB time and short physical performance battery (SPPB) score. Patients in the high SB group had significantly higher sitting SB time and lower SPPB scores than those in the low SB group.

Clinical characteristics between the high SB group and low SB group can be compared in Table 1, which shows that the high SB group comprised 47.6% (168 of 353) the patients. There were significant differences in employment, smoking, main diagnosis, HF as a comorbidity, ARB as a medication, and SB between the two groups. Total sitting SB time in the high SB group was higher than that in the low SB group (733.6 ± 155.3 vs 246.4 ± 127.4 min/day, *t* = − 32.03, p < 0.001; Table 1, Fig. 2). Further, the mean SPPB score in the high SB group was lower than that in the low SB group (10.5 ± 2.4 vs 11.2 ± 1.6 points, *t* = 3.26, p = 0.001; Table 1, Fig. 2).

**Table 1:** Clinical characteristics of the patients in the low SB and high SB groups.

Characteristic	High SB (n = 168)	Low SB (n = 185)	t**, χ^2^ value	p-value
Age (years)*	68.9 ± 14.0	70.3 ± 11.1	1.08	0.277
Sex, male (%)	127 (75.6)	140 (75.7)	0.00	1.00
Body mass index (kg/m^2^)*	23.9 ± 4.5	23.4 ± 4.1	− 1.14**	0.256
**Employment (%)**	85 (50.6)	119 (64.3)	6.80	0.012
Living together (%)	136 (81.0)	143 (77.3)	0.71	0.477
**Smoking (%)**	104 (61.9)	94 (50.8)	4.40	0.047
Marriage (%)	114 (67.9)	137 (74.1)	1.65	0.244
Main diagnosis (%)			18.00	< 0.001
Heart failure	66 (39.3)	39 (21.1)		
Ischaemic heart disease	89 (53.0)	113 (61.1)		
Valvular disease	6 (3.6)	12 (6.5)		
others	7 (4.2)	21 (11.4)		
Left ventricular ejection fraction (%)*	50.4 ± 14.7	50.3 ± 13.4	− 0.07**	0.943
Comorbidities (%)				
Hypertension	106 (63.1)	115 (62.2)	0.03	0.944
Diabetes	62 (36.9)	69 (37.3)	0.01	1.00
Dyslipidemia	95 (56.5)	107 (57.8)	0.06	0.891
** Heart failure**	72 (42.9)	59 (31.9)	4.54	0.043
Stroke	13 (7.7)	21 (11.4)	1.32	0.333
Renal dysfunction	45 (26.8)	35 (18.9)	3.11	0.102
Charlson comorbidity index*	2.3 ± 2.0	2.1 ± 2.2	− 1.00**	0.316
Haemoglobin (g/dL)*	13.0 ± 2.2	12.9 ± 2.2	− 0.50**	0.62
Creatinine (mg/dL)*	1.3 ± 1.5	1.3 ± 1.2	− 0.45**	0.651
Medications (%)				
Beta blocker (%)	121 (72.0)	137 (74.1)	0.18	0.757
ACE-I (%)	51 (30.4)	42 (22.7)	2.66	0.131
** ARB (%)**	37 (22.0)	62 (33.5)	5.76	0.023
Diuretic (%)	93 (55.4)	92 (49.7)	1.12	0.342
Statin (%)	119 (70.8)	115 (62.2)	2.96	0.108
Sitting SB (min)*	733.6 ± 155.3	246.4 ± 127.4	− 32.03**	< 0.001
SPPB (points)*	10.5 ± 2.4	11.2 ± 1.6	3.26**	0.001

*Mean ± standard deviation.

*SB* sedentary behaviour time, *ACE-I* angiotensin-converting enzyme inhibitor, *ARB* angiotensin II receptor blocker, *SPPB* short performance physical battery.

### Multiple linear regression analysis for total SPPB score

Table 2 shows the results of multiple linear regression analysis for the total SPPB score. The factors significantly associated with total SPPB score included all variables except for valvular disease as a main diagnosis, diabetes and renal dysfunction as comorbidities, and ARB as a medication in the univariate model. Moreover, in the multivariable model, the factors significantly associated with total SPPB score included age, sex, HF as a main diagnosis, marriage, and SB. The adjusted R^2^ value was 0.3962, and the F-statistic was 16.4 for the 15 variables in this analysis model.

**Table 2:** Multiple linear regression tests for total SPPB score.

Characteristic	Univariable model		Multivariable model	
	Beta (95% CI)	p-value	Beta (95% CI)	p-value
Age	− 0.077 (− 0.092 to − 0.062)	< 0.001	− 0.056 (− 0.074 to -0.038)	< 0.001
Sex	1.51 (1.04 to 1.98)	< 0.001	0.575 (0.113 to 1.04)	0.015
Body mass index	0.111 (0.063 to 0.160)	< 0.001	0.012 (− 0.035 to 0.058)	0.60
Main diagnosis				
Heart failure	− 1.90 (− 2.32 to − 1.47)	< 0.001	− 0.98 (− 1.70 to − 0.259)	0.008
Ischaemic heart disease	1.48 (1.07 to 1.88)	< 0.001	− 0.037 (− 0.720 to 0.645)	> 0.90
Valvular disease	0.298 (− 0.671 to 1.27)	0.50	− 0.048 (− 1.00 to 0.906)	> 0.90
Smoking	0.897 (0.478 to 1.32)	< 0.001	0.039 (− 0.360 to 0.438)	0.80
Marriage	1.17 (0.720 to 1.63)	< 0.001	0.91 (0.523 to 1.30)	< 0.001
Employment	1.76 (1.37 to 2.15)	< 0.001	0.038 (− 0.438 to 0.514)	0.90
Comorbidities				
Diabetes	− 0.483 (− 0.921 to − 0.044)	0.031	− 0.231 (− 0.591 to 0.129)	0.20
Heart failure	− 1.73 (− 2.14 to − 1.33)	< 0.001	− 0.382 (− 0.895 to 0.132)	0.14
Renal dysfunction	− 0.464 (− 0.97 to 0.043)	0.073	0.083 (− 0.333 to 0.498)	0.70
Stroke	− 0.815 (− 1.53 to − 0.098)	0.026	− 0.237 (− 0.832 to 0.359)	0.40
Medications				
ARB	− 0.251 (− 0.734 to 0.232)	0.30	− 0.159 (− 0.548 to 0.229)	0.40
Sitting sedentary behaviour time	− 0.001 (− 0.002 to 0.000)	0.004	− 0.001 (− 0.001 to 0.000)	0.017

*SPPB* short performance physical battery, *CI* confidence interval, *ARB* angiotensin II receptor blocker.

## Discussion

The present study presented novel findings. First, the high SB group comprised 47.6% of the patients, indicating that about half of patients with CVD tended to spend long hours in SB. Second, characteristics of the patients such as smoking, HF as a main diagnosis and as a comorbidity, but not employment or medications, were observed among the patients with CVD who had higher levels of SB. Third, patients in the high SB group participating in phase I CR had significantly lower SPPB scores than patients in the low SB group. Finally, multiple regression analysis revealed that the SB remained an explanatory variable for total SPPB score. These findings suggest that high SB is present in about half of patients even in phase I CR, and hence, new strategies should be developed to stimulate patients with CVD to encourage more movement and less sitting.

To our knowledge, this is the first report on the effects of SB in the present study population, although some studies have investigated the causes or frequency of SB and/or physical activity in hospitalized patients^4,10–13^. The rate of SB was high at 47.6% and is relatively close to that of a previous study (sitting out of bed, 43%)^12^, even though the assessment methods were different between the two studies. Previous studies have pointed out that elderly patients tend to have more sitting SB time and spend more time in bed^10,11^. Therefore, particular attention should be paid to elderly patients with high SB.

In terms of our study hypothesis, patients with CVD showed high SB compared to those with low SB, which may support the occurrence of adverse outcomes^4,14^. Previous studies also suggested that the pattern of accumulation of sedentary time is important and linked it to acute detrimental effects on vascular function, blood pressure and lipids, which may contribute to the risk for cardiovascular events and mortality^15,16,34,35^. There were significant differences in the characteristics of our study patients for employment, smoking, main diagnosis, HF as a comorbidity, and ARB as a medication between the two groups. It is interesting to note that these factors largely align with predictors of high SB levels in the general population and of disease^4,14–16,27,28,34,35^. The present findings suggested that these factors may impact high SB levels in patients with CVD.

The total sitting SB time in the high SB group was 733.6 min/day, which converts to approximately 12.2 h/day. A previous study reported that an objectively measured sitting SB time ≥ 9.5 h/day was associated with increased risk of all-cause mortality^36^. As well, another study also suggested that patients with CVD and pre-CR sedentary time of 10.4 h/day are at risk for the detrimental health effects of SB, making these individuals highly vulnerable to recurrent cardiovascular events and premature death^14^. Moreover, CR reduced SB in these patients by 0.4 h/day^14^. As there are differences in the measurement methods, term, and subjects between previous studies and the present study, it is difficult to compare them directly. SB primarily occurred during leisure time, when the patients were not participating in phase I CR, suggesting that leisure time sedentary activities during hospitalization should be specifically targeted to achieve the largest reduction in total sedentary time.

The mean total SPPB score was lower in the high SB group than that in the low SB group in the present study. It is important that early mobilization to reduce SB, early screening for sarcopenia, and aerobic exercise and resistance training during hospitalization be carried out during phase I CR. In a previous study, multivariate analysis showed pre-hospital walking level to be a strong influencing factor of low physical function as assessed by SPPB at hospital discharge^37^. The exclusion criteria of the present study included patients with ‘difficulty walking alone’, so this factor did not effect the results of our study.

A score on the SPPB of < 7 points was associated with a larger risk of the combined endpoints of hospitalization and mortality for any cause (odds ratio = 3.6, 95% confidence interval; 1.0–12.9, p < 0.05) in patients with acute HF^38,39^. In addition, another previous study revealed that the SPPB has a ceiling effect^40^, which may have affected the results of the present analysis. In addition, our exclusion of patients who could not walk alone, indicating that they probably had severe physical function pre-hospitalization, might have excluded patients with low levels of independence. This fact likely affected the results of the present analysis.

A previous study in post-acute cardiac patients suggested that an improvement in the total SPPB score of > 1 was identified as the minimum clinically important difference (MCID)^41^. As we did not examine improvement in the pre-post SPPB total score, we could not investigate MCID in phase I CR. However, the very important clinical finding was that the high SB group had significantly lower physical function. Therefore, given that patients with high SB will likely become physically disabled in the future, it may be necessary for staff in charge of phase I CR to intervene in patients with SB during hospitalization to improve their activities of daily living.

The multiple linear regression analysis identified the factors significantly associated with total SPPB score to be age, smoking, marriage, HF, valvular disease as the main diagnosis, and SB. In addition, the adjusted R^2^ value was 0.3962 in this analysis model, indicating that the independent variable explains about 40% of the middle-level variation in the dependent variable.

HF is the final common pathway of several etiologies such as ischaemic and valvular heart disease^42^. Thus, the high SB group might include patients who were on a pathway of advanced cardiac disease and were likely to reduce physical function, even if they showed independence at this admission. As indicated by the multiple regression analysis, even after adjustment for other factors, high SB can predict impairment as indicated by the SPPB score. As mentioned above, although hospital-associated disability occurs infrequently among hospitalized patients with acute HF, only 44% of patients were accepted into phase I CR^21^. The main barrier may be the lack of referral of patients to rehabilitation, which means that rehabilitation and its importance have not yet penetrated deeply into the mindset of clinicians. However, we could not investigate that the reason for this in the present study.

A hospitalized patient with high SB may be experiencing a decline in physical function as indicated by the SPPB. Therefore, it is important to address these behavioural changes in phase I CR by focusing not only on increasing physical function but also in reducing high SB.

### Study limitations

There are several limitations in the present study. First, this study included several cardiac diseases in the main diagnosis and comorbidities that require different treatments. Second, assessment of SB was by questionnaire and lacks objective measurements such as from an accelerometer. Third, domain-specific SB was not analysed in the present study and thus could not be investigated in detail. Because patients in phase I CR are, by definition, hospitalized, we could not investigate work or leisure time activities during hospitalization. Fourth, many patients were hospitalized for short-term procedures such as ablation or coronary angiography and were excluded from the final analysis, which may have led to the potential inclusion of selection bias in the present study. Fifth, the mechanism behind SB and other specific risk factors including, among others, cardiopulmonary function, hypertension, dyslipidemia, insulin resistance, obesity, reduced muscle strength and muscle activity, decreased vascular endothelial function, cognitive function, and neck and shoulder pain, were not investigated^43,44^. Nevertheless, the performance in both groups as indicated by the SPPB scores was close to optimal. Any one of these factors may have been a cause of the decline in physical function in the patients with high SB, as indicated by the lower SPPB score in this group. Finally, we could not investigate the relation between SB and prognosis such as rate of re-admission and mortality. Further studies are required to examine interventions to improve SB and SPPB. Despite these limitations, this multicentre clinical cohort study showed the effects of SB on physical function in phase I CR.

## Conclusions

**Figure 3:**
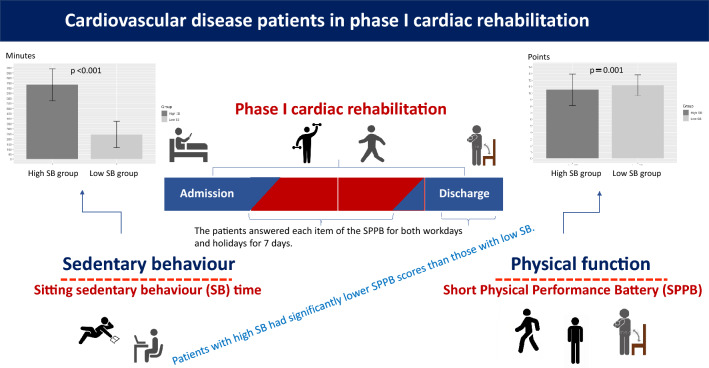
Graphic showing the importance of addressing sedentary behaviour to improve physical function. Effective strategies to improve physical function that take SB into consideration can be developed in phase I cardiac rehabilitation.

The relation between SB and SPPB as an index of physical function of patients with CVD in phase I CR was investigated. Patients in the high SB group had significantly lower SPPB scores than patients in the low SB group (Fig. 3). After adjustment for other associated factors in the multiple regression analysis, SB remained a factor associated with the total SPPB score. Our findings underscore the importance of addressing SB in patients with CVD even if they are undergoing phase I CR.

## Data Availability

The datasets used and/or analysed during the current study available from the corresponding author on reasonable request.
